# Autologous Bone Marrow Mononuclear Cell Transplantation in Patients with Decompensated Alcoholic Liver Disease: A Randomized Controlled Trial

**DOI:** 10.1371/journal.pone.0053719

**Published:** 2013-01-14

**Authors:** Laurent Spahr, Yves Chalandon, Sylvain Terraz, Vincent Kindler, Laura Rubbia-Brandt, Jean-Louis Frossard, Romain Breguet, Nicolas Lanthier, Annarita Farina, Jakob Passweg, Christoph D. Becker, Antoine Hadengue

**Affiliations:** 1 Division of Gastroenterology and Hepatology, University Hospitals and Faculty of Medicine, Geneva, Switzerland; 2 Division of Hematology, University Hospitals and Faculty of Medicine, Geneva, Switzerland; 3 Department of Radiology, University Hospitals and Faculty of Medicine, Geneva, Switzerland; 4 Division of Clinical Pathology, University Hospitals and Faculty of Medicine, Geneva, Switzerland; 5 Department of Bioinformatics and Structural Biology, University Hospitals and Faculty of Medicine, Geneva, Switzerland; Copenhagen University Hospital Gentofte, Denmark

## Abstract

**Objective:**

Impaired liver regeneration is associated with a poor outcome in patients with decompensated alcoholic liver disease (ALD). We assessed whether autologous bone marrow mononuclear cell transplantation (BMMCT) improved liver function in decompensated ALD.

**Design:**

58 patients (mean age 54 yrs; mean MELD score 19, all with cirrhosis, 81% with alcoholic steatohepatitis at baseline liver biopsy) were randomized early after hospital admission to standard medical therapy (SMT) alone (n = 30), including steroids in patients with a Maddrey’s score ≥32, or combined with G-CSF injections and autologous BMMCT into the hepatic artery (n = 28). Bone marrow cells were harvested, isolated and reinfused the same day. The primary endpoint was a ≥3 points decrease in the MELD score at 3 months, corresponding to a clinically relevant improvement in liver function. Liver biopsy was repeated at week 4 to assess changes in Ki67+/CK7+ hepatic progenitor cells (HPC) compartment.

**Results:**

Both study groups were comparable at baseline. After 3 months, 2 and 4 patients died in the BMMCT and SMT groups, respectively. Adverse events were equally distributed between groups. Moderate alcohol relapse occurred in 31% of patients. The MELD score improved in parallel in both groups during follow-up with 18 patients (64%) from the BMMCT group and 18 patients (53%) from the SMT group reaching the primary endpoint (p = 0.43 (OR 1.6, CI 0.49–5.4) in an intention to treat analysis. Comparing liver biopsy at 4 weeks to baseline, steatosis improved (p<0.001), and proliferating HPC tended to decrease in both groups (−35 and −33%, respectively).

**Conclusion:**

Autologous BMMCT, compared to SMT is a safe procedure but did not result in an expanded HPC compartment or improved liver function. These data suggest either insufficient regenerative stimulation after BMMCT or resistance to liver regenerative drive in patients with decompensated alcoholic cirrhosis.

**Trial Registration:**

Controlled-Trials.com ISRCTN83972743.

## Introduction

Decompensated alcoholic liver disease (ALD) in non-abstinent patients, often called alcoholic hepatitis, is a common cause of acute-on-chronic liver failure [1] with a poor outcome [2], [3]. This clinical syndrome of a recent onset of jaundice and/or ascites in a patient with ongoing alcohol misuse has been recently acknowledged in the EASL Clinical Practice Guidelines on alcoholic liver disease [4]. When available, liver biopsy will show steatohepatitis (ASH) in 70–90% of these patients [5]. The remaining 10–30% of decompensation episodes may be related to other causes, dominated by infection [6]. This clinical syndrome is also included in the definition of acute-on-chronic liver failure [1], [7] [8], in which patients’ outcome can be reliably assessed using severity scores including the MELD, a prognostic indicator initially created to predict survival after portal decompression by transjugular porto-systemic shunt [9]. The MELD score ranges from 6 (normal liver function) to 40 (severe liver failure). Although survival in severe, biopsy-proven ASH is improved by corticosteroids, significant mortality is observed in patients who are not eligible to steroids because of lack of access to transvenous liver biopsy and proven ASH, have “non-severe” ASH, or do not respond to steroids [2]. As no treatment has demonstrated superiority over steroids until now, and liver transplantation is not an option for most of these patients, alternative therapies are needed.

Impaired liver regeneration seems to be common to situations of ongoing liver failure. Clinically, prolonged periods of severe jaundice, low coagulation factors and clinically significant portal hypertension put these patients at risk of complications. On liver biopsies of patients with ASH, hepatic progenitor cells (HPC) expansion correlates with disease severity and mortality [10].

It has been proposed that pluripotent non-haematopoietic stem cells originating from the bone marrow may participate in the repopulation of a damaged liver and may improve its function [11]. Experimental studies in acute severe liver injury coupled with hepatocyte replication impairment suggest that regeneration derives partly from bone marrow cells and improves survival [12], [13]. Granulocyte colony-stimulating factor (G-CSF) mobilizes bone marrow cells and improves liver regeneration by stimulating oval cell proliferation and bone marrow cell engraftment in a rat model of liver injury [14]. We have demonstrated that G-CSF is well tolerated in patients with decompensated ALD and increases proliferative activity of both hepatic progenitor cells and mature hepatocytes in the short term [15]. A survival benefit has been recently demonstrated following repeated G-CSF administration in patients with acute-on-chronic liver failure, some of which were related to alcohol overuse [16]. The combination of mobilization, isolation, and direct infusion of bone marrow stem cells into the liver via the hepatic artery or the portal vein, or, alternatively, autologous human bone marrow stem cell transplantation, showed inconstant improvement in liver function. However, the majority of these trials have either included a limited number of patients, lacked appropriate control groups or were heterogeneous in their design and methods of stem cell therapy (reviewed in references [13], [17]). Therefore, tolerance, possible influence on liver fibrosis [18], potential benefits and mechanisms associated with bone marrow cell therapy in advanced liver disease and in particular in decompensated ALD characterized by an impaired liver regeneration [19], remain to be determined.

In order to test whether bone marrow cells mobilized and infused into the liver enhance hepatocyte proliferation and result in improved liver function over a 3-months period, we designed a pragmatic, prospective randomized trial in clinically decompensated ALD, comparing standard medical therapy (SMT) alone versus SMT combined with bone marrow mononuclear cell mobilization and infusion into the hepatic artery, reported as bone marrow mononuclear cell transplantation (BMMCT).

## Patients and Methods

### Patients

From February 2008 to March 2011, all patients admitted to our hospital for decompensated ALD were considered eligible if they met the following criteria: (i) clinical decompensation manifested by ascites and/or jaundice in active drinkers (≥80 g/day of alcohol); (ii) a liver biopsy performed within 7 days of admission; (iii) age 18 to 75 yrs; (iiii) MELD score <26; and (v) written informed consent to participate. Exclusion criteria included pregnancy, hepatitis B, C or HIV, documented hepatocellular carcinoma, biliary tract obstruction, liver biopsy showing causes other than ALD for decompensation, complete portal vein thrombosis, hypersensitivity to G-CSF, severe coagulopathy (platelets <50 G/l+INR >1.5), serum creatinine >150 umol/l, any ongoing infection, recent (<10 days) gastrointestinal bleed, estimated survival <6 months and clinically overt hepatic encephalopathy. High creatinine serum level and severe coagulopathy were considered exclusion criteria due to the risk of, a) further deterioration of renal function with the use of contrast media during angiography, and b) major bleeding during bone marrow aspiration and femoral artery catheterization.

## Methods

The protocol for this trial and supporting CONSORT checklist are available as supporting information; see Checklist S1 and Protocol S1.

### Design of the Trial

**Figure 1:**
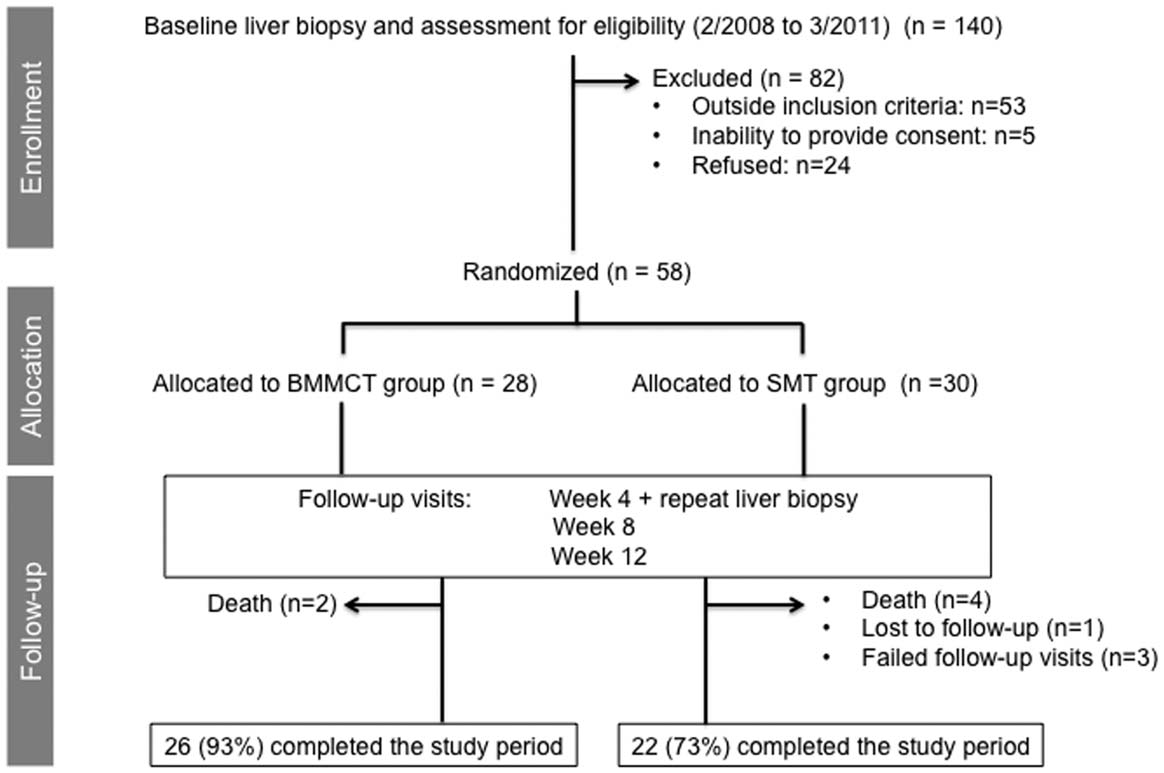
Flowchart of patients’ selection according to CONSORT guidelines and design of the study.

This randomized controlled trial (trial number: http://www.controlled-trial.com/ISRCTN83972743) was conducted in a single tertiary care centre (Geneva University Hospitals), and designed to compare SMT alone or combined with autologous BMMCT to improve liver function in decompensated ALD (figure 1 illustrates the flowchart according to CONSORT guidelines). Allocation to each treatment group was performed using a computer generated randomization code inserted in sequentially numbered opaque envelopes. The primary endpoint was an improved liver function at 90 days of follow-up, as defined by a decrease in the MELD score of at least 3 points as compared to baseline value. Based on our clinical experience, a decrease of at least 3 points from baseline MELD corresponds to a clinically relevant improvement in liver function and prognosis. To the contrary, an increase in MELD score within weeks of admission is associated with a poorer in-hospital mortality in patients with ASH [8]. The follow-up of 3 months was chosen based on the definition of acute-on-chronic liver failure [1] and on previous studies on bone marrow cell therapy [20]–[21] reporting improvement in liver function parameters during this observation period. Secondary endpoints included safety and evolution of parameters associated with liver regeneration and inflammation, including serum cytokines and liver tissues studies.

Shortly after hospital admission, all patients with decompensated liver disease suspected to be related to alcohol had a liver biopsy to confirm the diagnosis of ALD and to guide steroid therapy in cases of severe, histologically-proven ASH. Imaging studies were performed to rule out hepatocellular carcinoma and biliary tract obstruction. Particular attention was paid to detecting infection by cultivating ascites, urine, and blood, and by performing a chest radiograph, as recommended [4]. At time of randomization, peripheral blood samples were drawn for cytokines and blood markers of liver regeneration. Patients then received either SMT alone (including vitamin B supplements, stimulation of calorie intake, specialized support regarding alcohol abstinence but no pharmacological intervention, and a 4-week course of prednisone 40 mg/day in case of severe ASH, as defined by a Maddrey’s score ≥32 [2]) or SMT in combination with autologous BMMCT.

### Liver Tissue Studies

Both baseline and repeat liver biopsies were performed *via* the transjugular route under light sedation and hepatic hemodynamic measurements were performed as described [22]. In all cases, the size of liver biopsy specimen was sufficient for an accurate histological diagnosis.

#### Light microscopy

The liver biopsy specimen was immediately placed into formalin, then fixed and embedded, and processed for light microscopy. Serial sections 4 µm thick were stained with haematoxylin-eosin, reticulin and Masson Trichrome, and histological study was performed by an expert in liver disease (LRB) unaware of patients’ characteristics and treatment allocation. The entire biopsy specimen was examined by using 20–50 high-power fields (x 400) as previously described [15], [22]. Histological lesions common in ALD were analyzed in a blinded manner. Thus, we performed a semi-quantitative scoring of steatosis, portal and lobular inflammation, ballooning degeneration of hepatocyte, using the NAS score [23], but not fibrosis as all patients had cirrhosis. We also assessed the intensity of ductular reaction, thought to represent the compartment where hepatocyte and cholangiocyte progenitors expand in response to proliferation stimuli [24], using a semi-quantitative score (absent: 0; focal: 1; clusters <50% of microscopic fields: 2; clusters >50% of microscopic fields: 3).

#### Immunohistochemistry

We used a double immunohistochemistry staining method on liver tissue specimens to determine the number of proliferating cells in the ductular reaction [24]. To achieve this, we determined the number both of hepatic progenitors and intermediate hepatocytes according to morphological features and immunoreactivity against both cytokeratin 7 and the proliferation marker Ki67, as described [15], [25]. Immunohistochemical staining was performed in serial 3- to 5-µm-thick unstained sections from the tissue block, using the mouse monoclonal human antibody MiB1 against the proliferation marker Ki67 (1∶20 dilution) and the CK7 (1∶50 dilution) (Dako Cytomation, Zug, Switzerland). The preparation of slides was carried out as previously described [15]. Then, the whole biopsy specimen was analyzed under high magnification (x 400) and the HPC and intermediate hepatocytes that demonstrated double immunostaining were manually counted. Results are given as the mean number of cells per high-power fields.

### Cytokines and Blood Markers of Regeneration

We used a multiplex suspension array technique based on the sandwich immunoassay method (Bioplex, Biorad, Zürich, Switzerland) for multiple measurements on a single plasma sample [26]. This microsphere-based technology was applied for the dosage of TNFα (TNF), the soluble form of receptor-1 for TNFα (sTNFR1), and interleukin-6 (IL6), representative of inflammatory changes, and alfa-foetoprotein (AFP), hepatocyte growth factor (HGF) and transforming growth factor beta (TGFβ) as markers associated with liver [27], [28]. All measurements were performed on plasma samples obtained after immediate centrifugation of blood drawn in a fasting state, and immediately stored at -80°C until use. Cytokines concentrations were determined using commercially available immunoassays (R&D Systems, Abingdon, UK; Millipore, Saint Charles, MO, USA), with a detection limit of 5.5, 1.2, and 1.5 pg/ml, for TNF, sTNFR1 and IL6, respectively, and 40 pg/ml, 1.3 ng/ml, and 15.5 pg/ml for HGF, AFP, and TGFβ, respectively. Inflammation was therefore monitored using changes in TNF, sTNFR1 and IL6 plasma values, while liver regeneration was evaluated by the evolution of circulating levels of HGF, AFP as well as TGFβ as cytokines involved in hepatocyte proliferation [29].

### Bone Marrow Stem Cell Transplantation

Patients allocated to the BMMCT arm received a 5-day course of lenograstim (G-CSF, Granocyte, SANOFI Aventis, Meyrin, Geneva) to mobilize bone marrow cells at a dose of 10 µg/kg per day subcutaneously. On day 5 the bone marrow aspiration procedure was performed under propofol sedation. A mean volume of 103±18 ml of bone marrow was collected from the posterior iliac crest in bags containing 2500 IU heparin. Cell suspensions were immediately centrifuged (3000 rpm at 20°C for 7 minutes) over a Ficoll-Hypaque plus (GE-Healthcare, Uppsala, Sweden) gradient and mononuclear cells (MNC) recovered at the interface. Cells were washed twice with 140 mM NaCl containing 25% of hu-Albumin CSL 5% (Behring AG, Bern, Switzerland) and then suspended in 40 ml of human Albumin CSL 5% 1∶1 containing 10 U/ml of heparin, and transferred in a 400 ml infusion pouch. Analytical samples were taken to assess total nucleated cell count, haematopoietic colony forming unit (CFU) frequency, CD34+ cell frequency and mesenchymal stem cell (MSC) frequency. The CFU assay for MSC was read after 14 days of culture in methocult medium (Stem Cell Technologies, Grenoble, France), as previously described [30]. CD34 expression was performed on a FACScalibur (Beckton-Dickinson, Mountain View, CA) using the ISAGE dual platform protocol, and MSC frequency was determined after 10 days of culture by limiting dilution analysis using the Poisson statistical distribution. Due to the high viability rate of bone marrow MNC and the time schedule for autologous transplantation, no viability test was performed. The mean time between bone marrow aspiration and cell infusion was 5.9±1.2 hours. Under light sedation (propofol 20–50 mg IV), digestive angiography was performed by trained radiologists (ST, RB), and cells were slowly infused over 5 minutes into the proper hepatic artery through a 5F catheter (Terumo Glidecath, Leuven, Belgium). No contrast media was administered during or following cell infusion to avoid osmotic cell injury.

### Observation Period

The follow-up visits were scheduled at 1, 2 and 3 months after randomization. In addition to clinical and biological evaluations, alcohol consumption was monitored using blood ethanol levels at every follow-up visits. All adverse events were graded as mild, average or severe and classified according to Common Terminology Criteria for Adverse Events, version 4.0.

### Ethical Considerations

A written informed consent was obtained from each patient. The study protocol was in accordance with International Committee for Harmonization/Good Clinical Practice (ICH/GCP) and approved both by our institution Ethics Committee (Comité Départemental d’Ethique de Médecine Interne et Médecine Communautaire des Hôpitaux Universitaires de Genève, Protocole # 07–145) and by the Swiss regulatory authorities (SwissMedic, Institut Suisse des Produits Thérapeutiques, Bern, Switzerland, Protocole # 2008DR2031).

### Sample Size and Statistical Analysis

To achieve a statistical power of 85% at 5% type I error, with the initial assumption that the primary endpoint will be reached in 60% of BMMCT-treated patients and 20% in patients on SMT alone, a sample size of 26 patients in each arm was calculated. Assuming a 10% of included patients lost to follow-up, a sample of 60 patients was required. The assumptions we made for calculation of the sample size were based on data from several uncontrolled studies reporting significant improvement in liver function tests following bone marrow cell therapy [20] [31], and on the histological evidence of a sharp increase in hepatic progenitors proliferative activity early after G-CSF administration [15]. Patients who missed the 3-month follow-up visit, died or were transplanted were considered as not having reached the primary endpoint. Due to the non-parametric distribution of variables, data were expressed as mean and its standard deviation or median and its range. The Fisher’s exact test was used to compare the proportion of patients in each group reaching the primary endpoint. Binomial methods were used to construct the confidence intervals around percentages. Quantitative variables between groups were compared using the non-parametric Mann-Whitney *U*-test. The ANOVA repeated measure test with Bonferroni correction for multiple comparisons was used to assess MELD changes in each treatment group for the effect of time. Cytokines values and liver tissue parameters at baseline and at 3 months were compared using the Wilcoxon signed rank test. Correlations between MELD score changes and bone marrow cell subpopulations were made using the Spearman rank test. All analyses were based on the intention-to-treat principles. All tests were two-sided, and the level of significance was set at a p<0.05. The analyses were conducted using Statistical Package for the Social Sciences (SPSS 10.0 Chicago, IL, USA).

## Results

### Patients

The algorithm of patient selection complying with CONSORT Guidelines is provided in figure 1. The median time between admission and inclusion was similar between BMMCT and SMT groups (6 days [3]–[6] versus 6 days [3]–[22], p = 0.21, respectively). Table 1 provides the characteristics of the patients. At baseline biopsy, all patients had cirrhosis and steatosis, with hepatocyte ballooning present in all but 5 patients, and inflammatory changes in all but 3 patients. The definite histological criteria for ASH were reached in 47 (81%) of the patients, 22 in the BMMCT group (60% with a Maddrey’s score ≥32), and 25 in the SMT group (73% with a Maddrey’s score ≥32). In 11 patients, full criteria for ASH were not met as neutrophilic infiltration could not be evidenced on liver biopsy. Overall, all patients at baseline presented severe histological findings typically reported in acute, recent alcohol abuse [32]. The MELD score was similar between groups, showing liver insufficiency, and all patients presented with clinically significant portal hypertension. Due to severe ASH with Maddrey’s score ≥32, 17 patients and 22 patients received a 4-week course of prednisone in the BMMCT and SMT groups, respectively. The mean duration of hospitalization was 27 days [11–310] in the BMMCT group, and 27 days [8–212] in the SMT group (p = NS). Durable alcohol abstinence was achieved in 39 patients. However, 7 and 11 patients in the BMMCT and SMT groups, respectively, returned to moderate drinking (20–40 gr per week) in spite of motivational and psychological support delivered during the hospital stay. Except for one patient from the SMT group who was lost to follow-up, the outcome of all patients could be determined at 3 months.

**Table 1:** Baseline characteristics of the patients.

Characteristics	Variable	BMMCT groupn = 28	SMT groupn = 30	P value
Clinical				
	Age (yrs)	54 [34–66]	56 [37–68]	0.27
	Gender (M/F)	24/4	20/10	0.48
	Ascites (%)	72	60	0.29
	HVPG (mmHg)	19.1±2.8	19.9±2.3	0.46
Biological				
	MELD score	19±3.8	19.1±3.9	0.51
	Bilirubin (umol/l)	183±124	174±108	0.32
	INR	1.48±0.3	1.52±0.3	0.25
	Creatinine (umol/l)	67±21	68±23	0.18
	WBC (G/l)	9.5±5.2	6.6±2.9	0.17
	Albumin (gr/l)	22±4.6	23±4.8	0.19
Histological				
	Severe steatosis (%)	79	83	0.22
	Prominent hepatocyte ballooning (%)	54	57	0.20
	Marked inflammation (%)	68	57	0.26
	Definite ASH (n,%)	22 (79)	25 (83)	0.35
	Cirrhosis (%)	100	100	0.99

Abbreviations: HVPG: hepatic venous pressure gradient; WBC: white blood cell.

### Bone Marrow Stem Cell Transplantation

Laboratory procedures were successful in all patients, and the yield of mononuclear bone marrow cells was above 60% after Ficoll-Hypaque gradient centrifugation. The BMMCT procedure could be fully administered in all but 2 patients (one patient presented an acute variceal bleeding and another patient suffered from an aspiration pneumonia during the mobilization period by G-CSF). The mean number of MNC infused was 0.47±0.15×10^8^/kg, including 0.24±0.11×10^6^ CD34+ cells/kg and 3.39±5.9×10^5^ MSC/kg. No blood products were administered prior to arteriography. The infusion of MNC didn’t result in any thrombosis during or after the procedure.

### Liver Function Parameters

**Figure 2:**
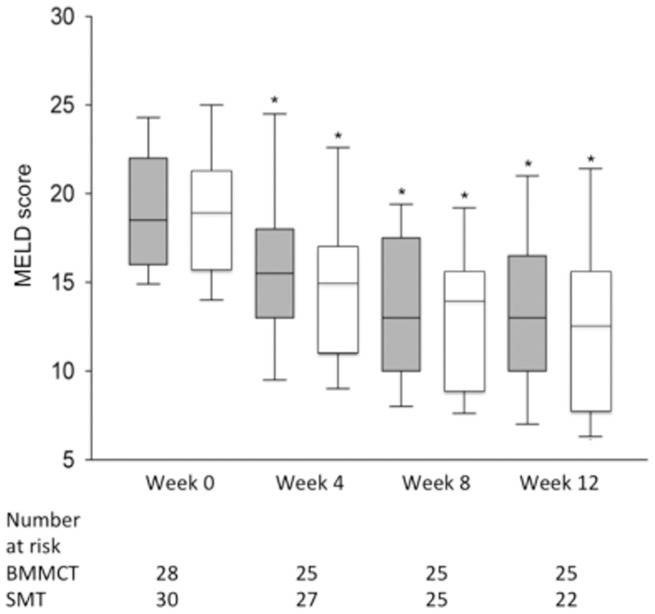
Box-plot (median value, lower and upper quartile) representation of the changes in MELD score during the 3-month follow-up period. The values of the BMMCT and SMT groups appear in grey and white, respectively. * p<0.05 versus baseline value.

**Figure 3:**
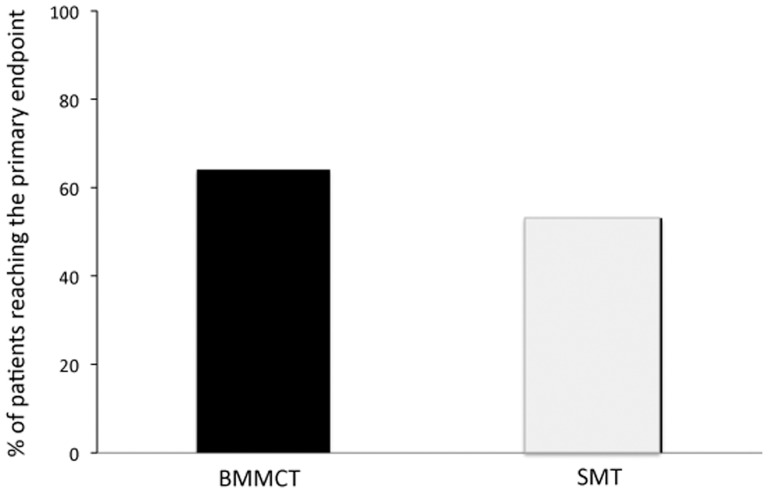
Percent of patients in each group who reached the primary endpoint at 3 months (p = 0.43).

The evolution of the MELD score showed similar improvement in both groups (figure 2). The percentage of patients who reached the primary endpoint was 64% in the BMMCT group and 53% in the SMT group (p value: 0.43 (OR 1.6, CI 0.49–5.4)(figure 3). In the subgroup of patients who maintained complete alcohol abstinence, the primary endpoint was reached in 81% and 74% in BMMCT and SMT groups, respectively (p = 0.17).

At 4 weeks, a reduction in the hepatic venous pressure gradient could be observed both in the BMMCT group (19.1±0.6 to 16.5±0.6 mmHg, p<0.01) and in the SMT group (19.9±0.4 to 18.2±0.6, p<0.03).

### Adverse Events

Adverse events were equally distributed between groups (Table 2). There were 17 serious adverse events including 2 deaths in the BMMCT group, and 24 serious adverse events in the SMT group associated with the death of 4 patients. Causes of death were related to acute variceal bleeding (day 35) and liver failure (day 75) in the BMMCT group, and to intracranial haemorrhage (day 15), sepsis (day 32, day 60), and multiple organ failure (day 51) in the SMT group. In one patient, a transient hematoma at the site of femoral artery puncture was considered a serious adverse event attributable to BMMCT procedure. With regard to the possible role of bone marrow cell in generating hepatocellular carcinoma, no liver cancer was detected during this short observation period.

**Table 2:** Safety data during follow-up.

Adverse event(grade)	BMMCT group(n, %)	SMT group(n, %)	p value
Mild	32 (84)	6 (16)	0.11
Average	35 (49)	36 (51)	0.24
Severe	18 (39)	28 (61)	0.16
**Serious adverse event**	17 (41)	24 (59)	0.13
Death	2	4	0.22
Infection	7	6	0.21
Hepatic encephalopathy	3	4	0.19
Variceal bleed	1	1	0.99
Other	6	13	0.17

### Liver Tissue Studies

The evolution of steatosis, hepatocyte ballooning and lobular inflammation in paired liver biopsies from 26 patients in each group is given in table 3. Steatosis decreased in both groups, but changes in ballooning degeneration and inflammation were not significant. As compared to baseline values, the number of proliferating HPC tended to decrease at 4 weeks both in the BMMCT (−35%, p = 0.06) and in the SMT group (−33%, p = 0.11) (see table 4).

**Table 3:** Liver tissue studies on paired liver biopsies (n = 52). Evolution of histological alterations on light microscopy.

	Histology	Baseline	4 weeks	P value
**BMMCT**	Steatosis	2.69±0.18	0.88±0.19	<0.001
	Inflammation	2.5±0.12	2.03±0.0	0.8
	Ballooning	1.42±0.6	0.81±0.8	0.06
**SMT**	Steatosis	2.6±0.21	0.5±0.1	<0.001
	Inflammation	2.43±0.15	2.15±0.11	0.7
	Ballooning	1.54±0.7	0.9±0.8	0.09

**Table 4:** Liver tissue studies on paired liver biopsies (n = 52). Evolution of proliferating hepatocytes at immunohistochemistry.

	Immunohistochemistry	Baseline	4 weeks	P value
**BMMCT**	Ki67+/CK7+ HPC	1.4±1.1	0.9±0.7	0.06
	Ki67+/CK7+ IH	0.3±0.6	0.3±0.4	0.89
**SMT**	Ki67+/CK7+ HPC	1.2±1.1	0.8±0.9	0.11
	Ki67+/CK7+ IH	0.2±0.2	0.2±0.3	0.61

Abbreviations: HPC: hepatic progenitor cell; IH: intermediate hepatocyte.

Note: results expressed as mean number of cells per high-power field.

### Cytokines and Blood Markers of Regeneration

**Figure 4:**
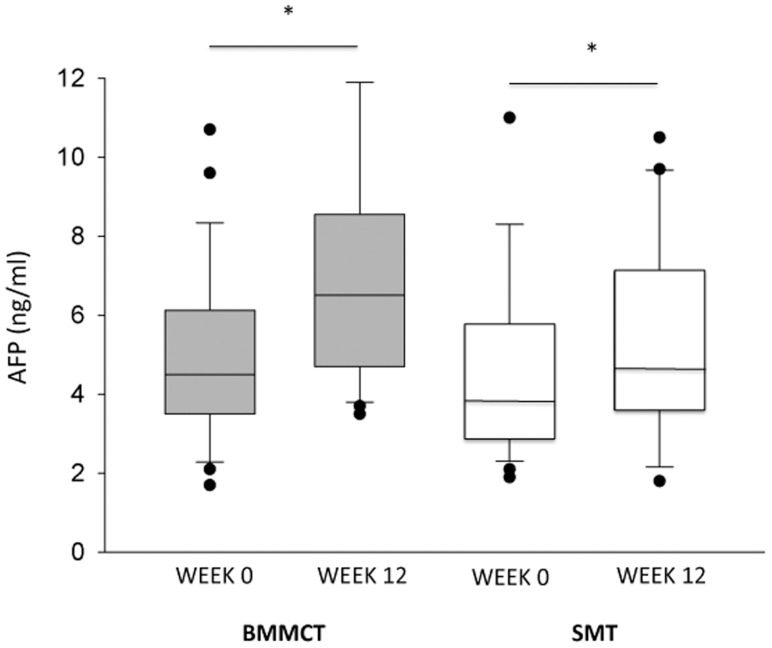
Box-plot (median value, lower and upper quartile) representation of AFP serum values in BMMCT (grey boxes) and SMT groups (white boxes) at baseline and at 3 months. * p<0.01 versus baseline.

**Figure 5:**
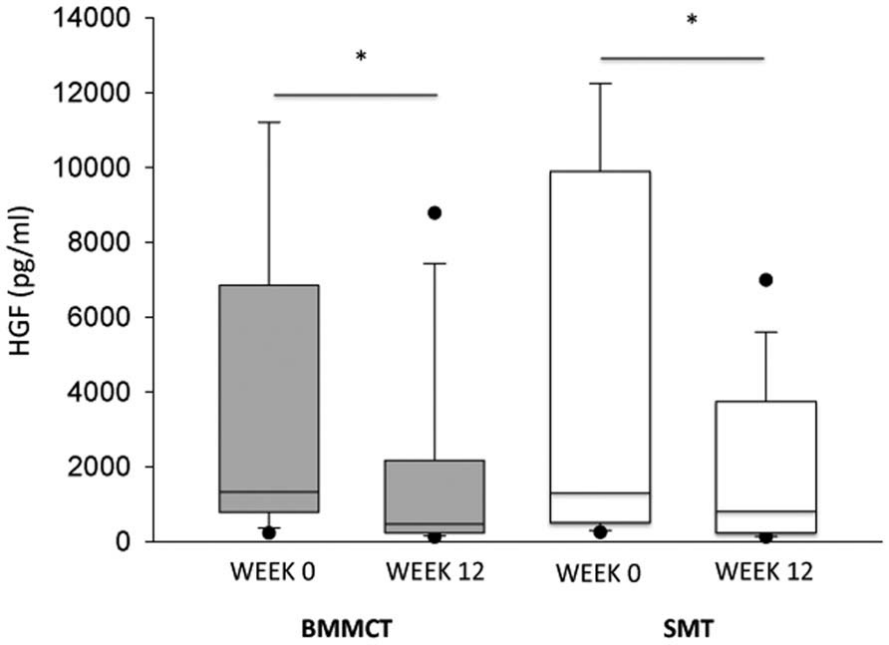
Box-plot (median value, lower and upper quartile) representation of HGF serum values in BMMCT (grey boxes) and SMT groups (white boxes) at baseline and at 3 months. * p<0.01 versus baseline.

**Figure 6:**
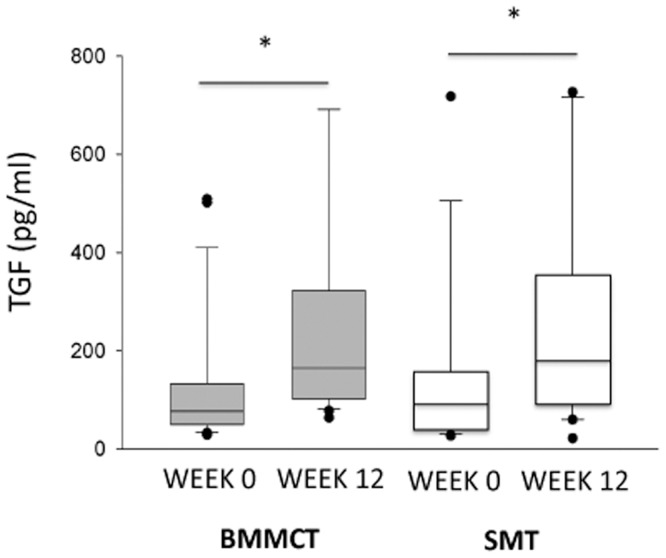
Box-plot (median value, lower and upper quartile) representation of TGFβ serum values in BMMCT (grey boxes) and SMT groups (white boxes) at baseline and at 3 months. * p<0.01 versus baseline.

Figure 4, 5, and 6, and table 5 provide data on markers of regeneration and inflammatory cytokines, respectively. Changes at 3 months in these biological parameters were similar in both groups. Patients who reached the primary endpoint had higher baseline HGF serum value as compared to those who didn’t (2870 [374–18318] versus 732 [248–12387] pg/ml, p<0.02).

**Table 5:** Changes in TNFα, sTNF-R1 and IL-6 serum concentrations at 3 months.

	BMMCT	
	Baseline	Week 12
TNFα (pg/ml)	8.34±2.64	7.93±2.64^§^
sTNF-R1 (pg/ml)	3960±1990	2560±1490^§^
IL-6 (pg/ml)	12.6±6	6.27±4.77^§^
	**SMT**	
TNFα (pg/ml)	8.34±1.93	7.36±1.47^§^
sTNF-R1 (pg/ml)	3890±2490	2390±1720^§^
IL-6 (pg/ml)	8.7±6.3	5.78±7.31^§^

Note: ^§^p<0.01 versus baseline.

### Correlations

In the BMMCT group, the changes in the MELD score at 3 months correlated neither with the total number of infused MNC (r = 0.2, p = 0.33), nor with the sub population of CD34+ cells (r = 0.05, p = 0.7), nor with MSC (r = 0.2, p = 0.33).

## Discussion

This trial was designed to test the potential of BMMCT to stimulate regeneration in patients with clinically decompensated ALD. In order to obtain mechanistic data on BMMCT on the liver, we repeated a liver biopsy at 4 weeks. We demonstrate that autologous BMMCT combined with SMT is not superior to SMT alone using an improvement in the MELD score as an endpoint.

This study was designed in accordance with the recommended approaches for trials of bone marrow stem cells in patients with chronic liver diseases [33]. In order to gain maximal benefit of BMMCT, we chose to combine G-CSF mobilization and direct administration to the diseased liver. Mobilization by G-CSF releases a moderate amount of CD34+ cells in blood [15], while an apparent small proportion of these cells participate in liver regeneration [11], [33]. The delivery of a higher concentration of bone marrow stem cells to the site of injury is thought to require either direct administration into the hepatic artery or the portal vein, or an *ex vivo* expansion of these cells prior to transplantation [17]. There is some concerns of using the portal vein with regards to the risk of infused cells bypassing the liver due to portal systemic collaterals present in advanced cirrhosis. To better ensure cell viability and feasibility in acutely ill patients, we excluded *ex vivo* expansion of cellular subpopulations, and selected the hepatic arterial route for stem cell reinfusion, as previously described in several studies [34] [20] [35]. Determining the fate of infused bone marrow cells is challenging. No clinical studies have explored their level of engraftment into the diseased liver parenchyma, partly due to the relative poor performance of cell tracing techniques available [17]. In addition, immunohistochemical expression of CD34, a marker of human hematopoietic stem cell, but also an endothelial cell marker [36], may be gradually lost several weeks following cell therapy.

We chose a 3-month follow-up with regards to the elevated morbidity and mortality reported in this patient population with decompensated ALD [2], [3]. There was a parallel evolution of the MELD score, biological values and liver tissue studies in both groups, showing a reduction in the number of proliferating HPC measured on the liver biopsy repeated at 4 weeks.

Following acute liver damage and liver cell loss, an increased number of proliferating hepatocytes on liver biopsy is associated with survival [37]. It is unknown whether bone marrow derived cells or therapeutic stimulation of mature hepatocytes and their progenitors may potentiate regeneration in ALD. We previously showed that G-CSF alone safely elicited mobilization of circulating CD34+ cells in patients with ASH, but without clinical improvement on the short term [15]. Others used bone marrow cell therapy in patients with liver failure related to hepatitis B without G-CSF, with a transient improvement in liver function [38]. Similar to bone marrow donation where large numbers of cells are needed, we chose to combine G-CSF-based mobilization with bone marrow aspiration. We isolated well characterized populations of MNC and hypothesized that direct autologous transplantation in the hepatic artery would constitute a sufficiently rich cellular stimulus for liver regeneration as a result of the combined proliferative effect of bone marrow cells and possible immune modulatory properties of MSC [17]. As all our patients had underlying cirrhosis, the reported risk of fibrosis promotion [39] was not considered to be significant in this situation.

Two lines of reasoning may account for the absence of benefit in our patients. Firstly, was the stimulus sufficient to induce proliferation at the site of injury? Secondly, are there any target organ specific features that would make it unresponsive to BMMCT? Although we didn’t expand the bone marrow derived cells in culture, the mean number of infused cells was in the range of previous studies [33]. Nevertheless, both CD34+ and MSC populations were lower than reported in healthy bone marrow transplant donors [40] leading one to speculate that the number of cells might have been insufficient to ensure adequate engraftment and stimulation of regeneration. Accordingly, improvement in liver function tests was observed following infusion of a greater number of mononuclear bone marrow cells (8×10^9^ cells as compared to_0.47×10^8^/kg in our study) in patients abstinent from alcohol with a moderately decompensated alcoholic cirrhosis [41]. In this study, increased activity in the liver area of 3 patients using indium-111 scintigraphy suggested significant cell engraftment. Thus, as the number of cells infused may be a critical parameter to translate into a measurable biological benefit, it is generally accepted that overall the proportion of bone marrow-derived hepatocytes repopulating a diseased liver is small [11] [17]. Other cellular subpopulations of the non haematopoietic bone marrow compartment include MSC possessing immune modulatory properties, endothelial progenitor cells [13] and macrophages. Their potential benefits [42] and caveats [33] in modulating liver repair mechanisms are still under investigation. In particular, concerns have been raised regarding both the profibrogenic potential of bone marrow cells and the risk of precipitating a malignant transformation of liver cells [17]. Therefore, optimal route of administration, fate, mechanisms of action and therapeutic potential of these bone marrow stem cells in regenerating a chronically diseased liver remain at present ill defined.

Our BMMCT protocol included a 5-day G-CSF course to mobilize bone marrow stem cell prior to bone marrow aspiration, a procedure reported to increase circulating HGF [43] and facilitate the homing of the cells in an animal model [44]. In patients with decompensated ALD with ASH, increased levels of circulating CD34+ cells were associated with proliferating HPC within days of G-CSF [15], and survival at 2 months was significantly improved in patients with acute-on-chronic liver failure [16]. The present trial, which combined G-CSF, isolation and concentration of bone marrow mononuclear cells, and direct reinfusion at the site of injury, was not able to demonstrate any effect on liver cell proliferation or improved liver function.

The second hypothesis relates to the target organ. Cirrhosis is a state characterized by replicative senescence of hepatocytes [45] that impairs liver cell. In alcoholic cirrhosis, acute and chronic exposition to alcohol interferes with liver regeneration [19], [46] and may impair expansion of HPC in response to liver injury. The florid alcoholic lesions present on early liver biopsy may create an unfavourable microenvironment for cellular replication in acutely decompensated ALD. In a randomized clinical trial on patients with cirrhosis of mixed etiologies, Lyra et al. reported an improvement in the Child-Pugh score but no significant changes in MELD score during 90 days following cell therapy [34]. In contrast, in patients hepatitis B, Peng et al. [38] reported a significant improvement in liver function for several weeks following autologous bone marrow stem cell transplantation. Thus, it may be hypothesized that the response to stem cell therapy might be influenced by the aetiology of liver disease, raising the possibility that the target organ might be unresponsive to the proliferative stimulus.

A third hypothesis for a lack of beneficial effects of therapy on liver function is alcohol relapse in 31% of our patients. Recurrence of alcohol misuse in this population is not rare, reported in approximately 40% of patients after a 3-month follow-up in a recent review [47]. However, the quantity of alcohol our patients declared during follow-up (20–40 gr/week) was clearly inferior as compared to the initial heavy consumption (80–100 gr/day) at time of hospital admission. Although a deleterious effect of this moderate amount of alcohol in liver function cannot be ruled out, MELD changes in the subgroups of abstainers and non abstainers were similar.

Our study has several strengths including well characterized study groups of acutely decompensated alcoholic cirrhotics with similar characteristics at baseline, including a balanced number of patients receiving steroids. However, we didn’t consider a treatment group receiving only G-CSF mobilization therapy in our study design. In fact, we chose to compare SMT alone to a strategy that we believed could be most efficient to promote liver repopulation and regeneration, that is G-CSF combined to autologous BMMCT. In addition, we were not able to study the fate nor the level of engraftment of the infused bone marrow cells into the liver parenchyma. Thus, whether a larger number of MNC are necessary to elicit a proliferative response in this clinical situation remains speculative. Finally, the immunostaining results obtained on the repeat liver biopsy provided only indirect information regarding the effect of treatment.

This trial in non-abstinent patients admitted with decompensated ALD is a truly negative study with comparable groups at baseline, without any tendency toward improvement in BMMCT-treated patients during follow-up. The parallel evolution of the MELD score, biological and liver tissue parameters including similar HPC profiles, suggest an apparent refractoriness to the proliferative stimulus. BMMCT, which included G-CSF mobilization therapy, failed to elicit HPC expansion and liver regeneration. Autologous bone marrow derived cell transplantation in patients with decompensated alcoholic cirrhosis is feasible and well tolerated, but cannot be considered as a suitable regenerative therapy. The mechanisms associated with this lack of benefit warrants further studies.

## Supporting Information

Checklist S1 CONSORT Checklist
(DOCX)

Protocol S1 Trial Protocol
(DOCX)
