# Supplementary material for: Autologous Bone Marrow Mononuclear Cell Transplantation in Patients with Decompensated Alcoholic Liver Disease: A Randomized Controlled Trial
Source: PLoS One. 2013 Jan 14;8(1):e53719. doi: 10.1371/journal.pone.0053719 (PMC3544843; doi:10.1371/journal.pone.0053719)
Supplement: Protocol S1 — Trial Protocol (DOCX) [file pone.0053719.s002.docx]

*PROJET DE RECHERCHE SOUMIS AU CENTRE DE RECHERCHE CLINIQUE (CRC)*

**Améliorer la fonction hépatique par transplantation autologue de cellules souches issues de la moelle osseuse lors de maladie alcoolique du foie décompensée : une étude contrôlée randomisée**

***Autologous human bone marrow stem cells mobilized by granulocyte colony-stimulating factor (GCSF) to improve liver function in patients with decompensated alcoholic liver disease: a randomized controlled study***

**Laurent Spahr1, Yves Chalandon2, Sylvain Terraz3, Laura Rubbia-Brandt4, Jakob Passweg2, Christoph Becker3, Antoine Hadengue1**

**1Gastroentérologie et Hépatologie, 2Hématologie, 3Radiologie, 4Pathologie Clinique, Hôpitaux Universitaires de Genève (HUG), Genève**

a) Résumé en français (200 mots)

L’hépatite alcoolique (HA) est une cause fréquente d’insuffisance hépatique et de décès (30% à 3 mois si forme grave). Le traitement est anti-inflammatoire (les stéroïdes), et devrait idéalement aider le foie à régénérer. Lors de cardiopathie ischémique, la stimulation de la moelle osseuse par le facteur de croissance GCSF mobilise des cellules souches pluripotentes dans le sang périphérique. Les cellules infusées dans les coronaires des territoires ischémiques améliorent la contraction myocardique. Dans le foie, des études animales montrent une meilleure régénération et survie après GCSF. Notre étude pilote chez des patients avec cirrhose et HA montrait une régénération active dans le groupe avec GCSF1. Le projet soumis ici (accepté par la Commission d’Ethique) consiste en une transplantation autologue de cellules souches de la moelle osseuse, infusées dans l’artère hépatique. C’est une étude contrôlée, randomisée, en collaboration avec 4 services issus de 3 départements des HUG. Le critère de jugement sera la régénération et l’amélioration de la fonction hépatique.

Une version précédente de ce projet soumis au CRC (septembre 2007) jugée trop préliminaire n’avait pas été retenue. Depuis, notre étude clinique avec le GCSF a été publiée (*Hepatology, juillet 2008*), et nous avons inclus 11 patients dans le projet soumis ici.

b) Scientific background

The spectrum of alcoholic liver disease includes alcoholic steatosis, the earliest lesion associated with alcohol abuse, alcoholic steatohepatitis (ASH) (often named alcoholic hepatitis), and cirrhosis2. Advanced liver disease, however, develops only in a minority of alcohol abusers. The risk of developing steatohepatitis is not linearly correlated with the amount of alcohol ingested. The pathogenesis is much more complex, including oxidative stress and release of inflammatory mediators. Patients with ASH represent a heterogeneous population. Histological features of steatohepatitis may be observed incidentally on the liver biopsy in the work-up of abnormal liver function tests, but more frequently patients come to medical attention at the time of liver-related complications, such as jaundice, ascites, variceal bleeding or hepatic encephalopathy, or with non-specific symptoms such as fatigue or vague abdominal discomfort.

Patients with alcoholic cirrhosis may be asymptomatic (compensated), or present with the above-mentioned symptoms (decompensated). Decompensation may result from factors that include alcohol abuse alone or associated with histological features of steatohepatitis, or infection.

**Prognosis**

The prognosis of patients diagnosed with ASH depends on the severity of the disease, which is best assessed by scores that include serum bilirubin and coagulation times alone (the Maddrey’s score) or in combination with serum creatinine (the MELD score, see Appendix). A change in the MELD score appears to be a reliable indicator of outcome and, if it decreases over time, a surrogate marker of survival. In our experience, a reduction in the MELD of > 3 points over a 2 to 3 months follow-up after decompensation is associated with a favourable outcome.

The serum level of the pro-inflammatory soluble receptor of TNF brings also important prognostic information3. In severe forms, the short-term mortality (i.e, at 3 months of diagnosis) is high, in the range of 40 to 50%. In non severe forms, there is a 10-15% mortality rate. Overall, in patients with ASH and decompensated liver disease, causes of death include renal insufficiency, infection or gastrointestinal bleeding resulting in liver failure. The characteristic features of patients who died include profound liver failure and inability to achieve efficient liver regeneration.

**Treatment**

The management of patients with ASH includes durable abstinence from alcohol, and initiation of a 28-day steroid course in severe, biopsy-proven disease. As recently demonstrated in a meta-analysis4, the 1-month survival is increased (85% versus 65%) in patients who received steroids. In spite of this, mortality remains elevated, either because on a non-response to steroids, infections, variceal bleeding, renal failure or liver insufficiency. To date, no treatment has demonstrated superiority over steroids. Although the first pilot trial using anti-TNF showed promising results5, a larger study failed to show a survival benefit is patients with ASH.

Patients with decompensated alcoholic cirrhosis are treated with supportive measures only. If liver failure persists following a prolonged period of abstinence, liver transplantation can be an option in selected patients. However, many patients with end-stage alcoholic liver disease are not candidate to liver transplantation due to their age or significant medical comorbidities.

To this day, despite numerous attempts to transplant isolated hepatocytes6, no strategy to improve liver regeneration capacity achieved clinically significant benefit.

**Bone marrow pluripotent stem cells**

The human bone marrow includes two types of stem cells, haematopoietic stem cells (HSC) that support haematopoiesis, but also non-haematopoietic stem cells of mesenchymal origin that possess a multilineage potential7. The latter are referred to as bone marrow-derived stromal cells or mesenchymal stem cells (MSC) and are capable of differentiating into both mesenchymal and non-mesenchymal lineages. Bone marrow (BM) cells have been shown to differentiate into hepatocytes and intestinal cells through the detection of Y chromosome- containing cells in sample from female recipients of BM cells from male donors. BM transplantation has long been used to treat a variety of haematological diseases through haematological reconstitution via haematopoietic stem cells. BM cells are of interest because they are easily isolated from a small aspirate of bone marrow and can be transplanted in an autologous manner into a diseased organ for cellular repopulation and repair. Pre-clinical studies in animal models of liver failure showed the potential of BM cells therapy to repopulate the damaged liver and to differentiate into albumin-producing hepatocytes, increase serum albumin levels, reduced liver fibrosis8, and improved survival rate. This has led the same group to treat 9 patients with liver cirrhosis with autologous bone marrow transplantation (ABMT) via infusion of BM cells harvested under general anaesthesia through a peripheral vein. This was well tolerated, and associated to a moderate improvement in serum albumin and Child-Pugh score, although the exact mechanism was not elucidated9. MSC-based cell therapy has also been tested in ischemic heart disease. Intracoronary injection of bone marrow stem cells or MSC has been shown to be a successful approach to treat a dyskinetic myocardial territory. Chen et al.10 carried out a clinical study in which 60 ml of bone marrow were aspirated from the iliac crest, and mononuclear cells separated and cultured, and then injected into the target coronary artery. In the treated patients, there was a significant reduction in the dyskinetic areas of the myocardium at 3 months of follow-up.

On the other hand, HSC are better characterized and belong to the CD34+ CD133+ cell population. They give rise to all lineages of blood cell differentiation. The advantage is that they can be prospectively isolated from haematopoietic tissue in known numbers. Granulocyte colony-stimulating factor (G-CSF) is a well-known stem cell mobilizer, commonly used in a clinical practice to increase the level of circulating haematopoietic progenitors in the setting of bone marrow donation. Administration of G-CSF to patients with cirrhosis is safe and followed by an increased levels of CD34+ stem cells, although this effect is blunted as compared to healthy subjects. A 5-day course of G-CSF 5 g/kg bid was followed by an increased proliferation of hepatocyte lineage cells (using double MIB-1/CK7 or CK18 immunostaining) in patients with ASH1, but this did not translate into an improved liver function over the 28 days of the study. The approach of direct infusion of SC at the site of tissue injury (after mobilization by G-CSF) has demonstrated superior efficacy as compared to mobilization alone by G-CSF in a large prospective study in ischemic heart disease11.

There is no randomized study published of treatment with SC in patients with end- stage liver disease. However, a phase I study performed to determine the safety and tolerability of injecting autologous CD34+ cells (i.e. haematopoietic stem cells) in a small number of patients with liver insufficiency was recently published12. Following G-CSF administration, 106 to 108 CD34+ cells were injected into the portal vein (in 3 patients, under CT scan) or into the hepatic artery (in 2 patients, via arteriography). Both procedures were well tolerated, and liver function tests apparently improved. This was also the case in the other study mentioned above with autologous BM infusion infused in a peripheral vein. Thus, autologous bone marrow infusion appears promising and safe to improve the function of a diseased organ.

Based on those pre-clinical and clinical studies we propose here to combine both approaches of stimulating peripheral HSC with G-CSF and transplanting these cells directly into the hepatic artery in an autologous manner hoping that combining HSC and MSC will synergise and translate into a better outcome in patients. Indeed, similar studies in BMT patients have shown that BM harvest stimulated with G-CSF contained a higher CD34+ count and more granulocyte-macrophage colony-forming units (haematopoietic progenitors).

b) Aim of the study

1. To demonstrate an improvement of liver function as assessed by the MELD score following GCSF-mobilized autologous bone marrow stem cells embolized into the hepatic artery

2. To study the mechanisms that underly the beneficial effects of pluripotent bone marrow stem cells on the liver parenchyma

***Primary endpoint***

The primary endpoint will be the improvement of liver function, as assessed by a decrease in the MELD score of > 3 (delta MELD) between baseline, day-28, day-60, and day-90 follow- up visits.

***Secondary endpoints***

Secondary endpoints will include: improvement in liver function as assessed by the following parameters (serum bilirubin; serum albumin; coagulation time (PT and INR); ascites; hepatic encephalopathy) which allow the calculation of the Child-Pugh’s score at day 28, day 60, and day 90; mortality at 3- and 6 months of follow-up; evolution of serum markers of liver regeneration (alpha-foetoprotein: AFP, hepatocyte growth factor: HGF), inflammation (soluble receptor-1 of TNF : sTNF-R1, TNF: interleukin-6: IL-6) and fibrosis (transforming growth factor : TGFβ); changes in liver histology at day 28

**Design of the study**

This study is a prospective randomized controlled study performed in a single tertiary care centre with expertise in haematology, interventional radiology, pathology and hepatology. The inclusion period will be 24 months. Patients with decompensated alcoholic liver disease will be randomized to receive either standard medical care (including corticosteroids if indicated) only or associated to autologous stem cells isolated from the bone marrow following a 5-day course with GCSF and then embolized into the hepatic artery.

In patients hospitalized with decompensated alcoholic liver disease, the standard of care includes routine laboratory values, imaging of the liver by ultrasonography and computed tomography, as well as a liver biopsy. Biological parameters required to calculate the MELD (and the Child-Pugh’s score) will be obtained at the time of liver biopsy. In our experience, the median time between hospital admission and liver biopsy is 4 days. After the consent form is signed, included patients randomized to stem cell therapy will receive a 5-day course of GCSF (10 mcg/kg daily x 5 days) followed by a 60 ml bone marrow aspiration from the iliac crest under local anaesthesia. Then, cells (a majority of CD34+, and some mesenchymal/mononuclear cells) will be isolated from the aspirate using a classical Ficoll density separation. Due to the high viability rate of these SC, the injection procedure will be performed within 36h of bone marrow aspirate. The injection procedure will take place as follows: the suspension of cells (approximately 0.5 x 108 cells, with a total volume of 30-50 ml) will be selectively embolized via arteriography into the right and left hepatic artery branches, so as to distribute CD34+/MC in both lobes of the liver. To minimize the risk of cellular alterations during the intra-arterial infusion, the embolization procedure will not be followed by the use of contrast media, unless medically required. Patients randomized to control group will receive standard medical care only. Circulatory cytokines involved in regeneration (AFP, HGF), inflammation (sTNF-R1, TNF, IL-6), and fibrosis (TGFβ) will be measured in both groups at baseline, day 28, day 60, and day 90 visits.

At day 28, patients will be readmitted to the day care hospital for a repeat liver biopsy and assessment of routine laboratory tests (coagulation, haematology and liver function tests) and cytokines. Follow-up visit will also include a careful record of all clinical events and current medications, as well as a physical examination. Due to the key role of sérum creatinine in the determination of the MELD score (see Appendix), particular attention will be paid to the dose of diuretics administered to included patients.

**Inclusion criteria**

**Inclusion** Age 18-75 yrs; biopsy-proven alcoholic liver disease; abnormal liver function (MELD 10-26); written informed consent **Exclusion**  Recent (10 days) infection or digestive haemorrhage; estimated survival < 6 months; coexistent HIV, hepatitis B or C infection; portal vein obstruction (main trunk or right/left branch); documented hepatocellular carcinoma; severe liver atrophy; baseline blood leucocytes > 25G/L; known hypersensitity to GCSF; renal failure (serum creatinine > 150 mol/L); contraindication to GCSF subcutaneous administration or arteriography (platelets < 50 G/L + INR > 1.5); clinically overt hepatic encephalopathy that may interfere with the informed consent; absence of written consent

**Sample size calculation**

To achieve a statistical power of 85% at 5% type I error, with the initial assumption that the MELD response (reduction > 3) will be obtained in 60% of the treated patients and 20% in the non treated patients, a sample size of 26 patients in each group was calculated. Assuming also that 10% of included patients in each arm will be lost to follow up, the sample size will be 30 patients in each arm.

**Methods**

Imaging Computed tomography (CT) will be performed in both groups, in order to obtain liver

volumetry, to describe arterial anatomy of the liver and to identify any unsuspected complication of the advanced alcoholic liver disease. The initial CT is part of the standard care of a patient with decompensated liver disease.

Imaging will be performed with a 64-detectors CT scanner (Siemens, Erlangen, Germany) with the following parameters: 2 ml/kg of iodinated contrast agent (Iohexol 350, GE Healthcare), rate of 3–5 ml/s, bolus tracking, section thickness 2.0 mm, reconstruction interval 1.0 mm. Multiphasic (arterial, portal and delayed) contrast-enhanced scans will be obtained during inspiration and will cover the whole liver parenchyma. A software (OsiriX) will determine the volumetry on axial images by manual delineation; the liver contours, the falciform ligament and the hepatic veins will be used as guides. The volumes of right and left hepatic lobes, segment 4 and total liver parenchyma will be calculated.

Four weeks after stem cells embolization or standard medical care, the same CT protocol will be applied in all patients. Volumetry of the liver will be determined according to the same parameters.

G-CSF administration Lenograstim (G-CSF) is a human granulocyte colony-stimulating factor produced by recombinant DNA technology. Absorption and clearance follows a first-order pharmacokinetic modeling without apparent concentration dependence. Patients allocated to the treatment group will receive G-CSF (lenograstim) 10 mcg/kg scut per day starting within 24 hours of liver biopsy, with a total mobilization period of 5 days. Commercially available vials contain 105 mcg and 263 mcg. For patients with a body weight up to 80 kg, doses will be 263 mcg vials twice in the morning, and 263 mcg once in the evening. For those with a body weight over 80 kg, doses will be 263 mcg twice in the morning, and 263 mcg twice in the evening. In a recent pilot trial using filgrastim (another commercially available human granulocyte colony-stimulating factor) in patients with cirrhosis and liver failure, this dosage was well tolerated and associated with a significant increase in circulating CD34 positive cells, although to a lesser extent as compared to healthy bone marrow donors.

**Liver biopsy** The liver biopsy is a key step in the initial work-up of a patient admitted for

decompensated alcoholic liver disease. This procedure, when performed by the transjugular route, is available within days of hospitalization, and is considered as a safe procedure in experienced operators’ hands. The liver biopsy will be repeated at day 28 in both treatment arms. In our institution, such a repeat procedure is well accepted in patients with cirrhosis13. Studies in liver tissue specimens will include standard haematoxylin-eosin staining, as well as MIB1 Ki67 immunostaining using monoclonal antibody (to assess the intrahepatic cell proliferative activity). The pathologist expert in the field of liver diseases (L.R-B) will be blinded to patients’ characteristics and treatment allocation.

**Biological values**

***Cytokines*** Blood will be drawn in sterile conditions in heparin-coated tubes and in a fasting state at baseline, and at follow-up visits at day 28, 60 and 90. After centrifugation at 3000 rpm for 15 minutes, aliquots of serum will be stored at -70°C until assayed. Commercially available immunoassays will be purchased, and measurements made according to the manufacturer’s recommendations. The following cytokines will be measured: TNF, sTNF-R1, IL-6, AFP, HGF, TGFβ(the latter is involved in the regulation of both hepatocyte and hepatic progenitor cells).

***Hematology*** Complete blood count (including platelet count, recently shown to be involved in the regeneration processes) will be performed at baseline as part of initial work- up. Blood leucocytes will be measured at day 5 of the mobilization course. Complete blood count determination will be repeated at day 28, day 60 and day 90 visits.

***Coagulation studies*** INR, prothrombin time, factor V levels will be measured at baseline (as part of initial assessment of liver function parameters). Values determined at days 28, 60 and 90 will be part of the study protocol measurements.

***Blood chemistry*** Electrolytes, urea, serum creatinine, CRP, and liver function tests, and serum albumin will be measured at baseline as part of initial evaluation of liver function. Then, all parameters will be measured again at day 28, 60, and 90 follow-up visits.

**Hepatic artery catheterization + embolization procedures** Catheterization of the hepatic artery will take place within 36 hours of bone marrow aspiration, using a classical arteriographic approach. A 5F introducer will be placed in the right or left common femoral artery. Diagnostic visceral arteriography (including celiac trunk and superior mesenteric artery) will be performed with a 5F catheter, in order to determine the presence of variant arterial anatomy and to confirm patency of the portal vein. Then, the catheter will be advanced into the right (distal to the cystic artery) and left hepatic artery, and into all accessory arteries of the liver if present. Non-ionic and iso-osmolar contrast media (iopamidol 300) will be used to guide the injection. If vasospasm occurs, arterial vasodilators (lidocaine hydrochloride 1% or papaverin) will be used. The aim of the procedure will be to deliver the entire dose of suspension of cells into the whole liver. The suspension will be infused with a volume of distribution into each artery that is proportional to the territories of their tributaries. The catheter will then be removed and haemostasis achieved by manual compression. The embolized arteries will be noted and the amount of suspension of cells injected into them will be recorded. All complications and side effects observed during the procedure will be carefully recorded.

**Potential impact on patients care**

In the view of the promising results regarding hepatocytes proliferation obtained in our pilot study on patients with cirrhosis and ASH1, we believe that performing an autologous transplantation of GCSF-stimulated bone marrow pluripotent stem cells into the liver will improve regeneration and function. If this hypothesis is correct, this therapeutic approach may be proposed to many patients with advanced alcoholic liver disease who are not candidate to liver transplantation.

**Statistics**

Due to the non parametric distributions of variables, data will be expressed as median and ranges. All analyses will be based on the intention-to-treat principles. Changes in MELD scores at day 0 and day 90 will be expressed as delta changes. The comparison between changes in the treated- and non treated patients will be performed using the non parametric Wilcoxon signed rank test with Bonferroni corrections for multiple comparisons. The Chi-square test will also be used to compare the proportion of patients in each group who achieved the > 3 reduction in MELD score. The Kaplan-Meier method with the log-rank test will also be used to compare differences in the rates of primary endpoint achievement at day 28, 60 and 90 after randomization. All data analysis will be conducted using a SPSS statistical software. A p value < 0.05 will be considered statistically significant.

**Appendix**

MELD score: The MELD score (Model for End.Stage Liver Disease) is currently used in a number of chronic liver diseases. The model is based on only objective variables that are readily obtained. It includes the coagulation function (using the INR, not subject to inter- laboratory variations as for the prothrombin time), the serum bilirubin (a good integrator of liver insufficiency) and the serum creatinine as a measure of renal function, a well-recognized predictor of survival in patients with liver diseases. The original mathematical formula for MELD is: MELD = 9.57 x log (creatinine) + 3.78 x log (serum bilirubin) + 11.2 x log (INR) + 6.43. The score is easily calculated on handheld computing deviced) Timing

**References**

1. Spahr L, Lambert J-F, Rubbia-Brandt L, Chalandon Y, Frossard JL, Giostra E, Hadengue A. Granulocyte colony-stimulating factor (G-CSF) induces proliferation of hepatic progenitor cells in alcoholic steatohepatitis: a randomized trial. Hepatology 2008;48:221-9.

2. Spahr L, Hadengue A. Alcoholic liver disease; natural history, diagnosis, clinical features, evaluation, prognosis and management. In: Rodes J, Benhamou J.P, Blei A, Reichen J and Rizzetto M, eds.Textbook of Hepatology; From basic Science to Clinical Practice. Volume 2. Oxford: Blackwell Publishing 2007: 1157-1178

3. Spahr L, Giostra E, Frossard JL, Bresson-Hadni S, Rubbia-Brandt L, Hadengue A. Soluble TNF-R1, but not tumor necrosis factor alpha, predicts the 3-month mortality in patients with alcoholic hepatitis. J Hepatol 2004;41:229-34.

4. Mathurin P, Mendenhall CL, Carithers RL, Jr., Ramond MJ, Maddrey WC, Garstide P, Rueff B, Naveau S, Chaput JC, Poynard T. Corticosteroids improve short-term survival in patients with severe alcoholic hepatitis (AH): individual data analysis of the last three randomized placebo controlled double blind trials of corticosteroids in severe AH. J Hepatol 2002;36:480-7.

5. Spahr L, Rubbia-Brandt L, Frossard JL, Giostra E, Rougemont AL, Pugin J, Fischer M, Egger H, Hadengue A. Combination of steroids with infliximab or placebo in severe alcoholic hepatitis: a randomized controlled pilot study. J Hepatol 2002;37:448- 55.

6. Fox I, Roy-Chowdhury J. Hepatocyte transplantation. J Hepatol 2004;40:878-886.

7. Preston SL, Alison MR, Forbes SJ, Direkze NC, Poulsom R, Wright NA. The new

stem cell biology: something for everyone. Mol Pathol 2003;56:86-96.

8. Sakaida I, Terai S, Yamamoto N, Aoyama K, Ishikawa T, Nishina H, Okita K.

Transplantation of bone marrow cells reduces CCl4-induced liver fibrosis in mice.

Hepatology 2004;40:1304-11.

9. Terai S, Ishikawa T, Omori K, Aoyama K, Marumoto Y, Urata Y, Yokoyama Y,

Uchida K, Yamasaki T, Fujii Y, Okita K, Sakaida I. Improved liver function in patients with liver cirrhosis after autologous bone marrow cell infusion therapy. Stem Cells 2006;24:2292-8.

10. Chen SL, Fang WW, Ye F, Liu YH, Qian J, Shan SJ, Zhang JJ, Chunhua RZ, Liao LM, Lin S, Sun JP. Effect on left ventricular function of intracoronary transplantation of autologous bone marrow mesenchymal stem cell in patients with acute myocardial infarction. Am J Cardiol 2004;94:92-5.

11. Kang HJ, Kim HS, Koo BK, Kim YJ, Lee D, Sohn DW, Oh BH, Park YB. Intracoronary infusion of the mobilized peripheral blood stem cell by G-CSF is better than mobilization alone by G-CSF for improvement of cardiac function and remodeling: 2-year follow-up results of the Myocardial Regeneration and Angiogenesis in Myocardial Infarction with G-CSF and Intra-Coronary Stem Cell Infusion (MAGIC Cell) 1 trial. Am Heart J 2007;153:237 e1-8.

12. Gordon MY, Levicar N, Pai M, Bachellier P, Dimarakis I, Al-Allaf F, M'Hamdi H, Thalji T, Welsh JP, Marley SB, Davies J, Dazzi F, Marelli-Berg F, Tait P, Playford R, Jiao L, Jensen S, Nicholls JP, Ayav A, Nohandani M, Farzaneh F, Gaken J, Dodge R, Alison M, Apperley JF, Lechler R, Habib NA. Characterization and clinical application of human CD34+ stem/progenitor cell populations mobilized into theblood by granulocyte colony-stimulating factor. Stem Cells 2006;24:1822-30.

13. Spahr L, Rubbia-Brandt L, Pugin J, Giostra E, Frossard JL, Borisch B, Hadengue A.

Rapid changes in alcoholic hepatitis histology under steroids: correlation with soluble intercellular adhesion molecule-1 in hepatic venous blood. J Hepatol 2001;35:582-9.
